# Use of a Bipedicled Pericranial Flap and a Split Thickness Skin Graft for Reconstruction of a Traumatic Scalp Injury: A Case Report

**DOI:** 10.7759/cureus.29887

**Published:** 2022-10-03

**Authors:** Hesham Alokaili, Tuqa A Alsinan, Duaa Almansour, Tareg M Alhablany, Ebtisam Alhuwaider, Felwa A AlMarshad, Tanveer A Bhat, Anas Aljasir

**Affiliations:** 1 Department of Plastic and Reconstructive Surgery, King Saud Medical City, Riyadh, SAU; 2 Department of Plastic and Reconstructive Surgery, Qatif Central Hospital, Qatif, SAU; 3 Plastic and Reconstructive Surgery Section, Department of Surgery, King Faisal Specialist Hospital and Research Centre, Riyadh, SAU

**Keywords:** split-thickness skin graft, reconstructive surgery, scalp reconstruction, scalp injury, bipedicled pericranial flap

## Abstract

Reconstruction of a scalp defect should ensure the skull’s protection, soft-tissue bulk, and contour maintenance. When calvaria is exposed, each reconstruction option has its own advantages and disadvantages. We report a 2-year-old Saudi boy, a road traffic accident (RTA) victim, otherwise medically stable who sustained partial to full-thickness defects of the scalp involving the left temporoparietal region, measuring 20 × 10 cm^2^ in size. After optimal debridement of the wound, a bipedicled pericranial flap with a split-thickness skin graft (STSG) was done. This case reports the satisfactory outcomes of using a bipedicled pericranial flap with STSG in traumatic scalp injuries, specifically in the pediatric age population without creating any secondary scalp skin defect and its associated morbidities. Being bipedicled the vascularity of the flap is more reliable and robust.

## Introduction

Protection of the cranium is a priority to prevent desiccation or infectious complications. The valveless head and neck venous system renders emissary and facial veins as a pathway for transmitting infection from soft tissue to the central nervous system [1]. The blood supply to the scalp is generous to such a degree as to exempt it from some conventions. For example, some authors avoid debridement of devitalized tissue due to the possibility of recovery in borderline tissues [1]. Regarding calvaria denuded of pericranium, pericranial flaps are well fit for calvarial coverage due to their proximity, reliable vascularity, and limited donor site morbidity [2]. These flaps may be raised in bipedicle or unipedicular fashion [3]. Its blood supply reflects the scalp, which is split into four vascular territories [3]. Anteriorly by supraorbital and supratrochlear arteries; lateral supply by the superficial temporal artery which divides into parietal and frontal branches at the superior most level of the external ear; posterolaterally by the posterior auricular artery; and posteriorly by occipital arteries and muscular perforators [1,3]. However, the dissection of subcutaneous tissue should be judicious to minimize nervous tissue disruption. The parietal region is especially advantageous due to its mobility as a function of gliding over smooth temporal fascia [1]. The aim of this paper is to display a rare flap in the reconstruction ladder of scalp defects which is devoid of creating secondary donor site scalp skin defect and its associated morbidities besides being a reliable and robust treatment option for traumatic scalp injuries. We believe the usage of pericranium flaps and skin grafting reduces blood loss, time under anesthesia, and donor site morbidity when compared to other methods such as locoregional cutaneous flaps or free tissue transfer. In addition, this method preserves existing hairlines and hair orientation to a greater degree than when compared to larger local flaps with a cutaneous component.

## Case presentation

**Figure 1:**
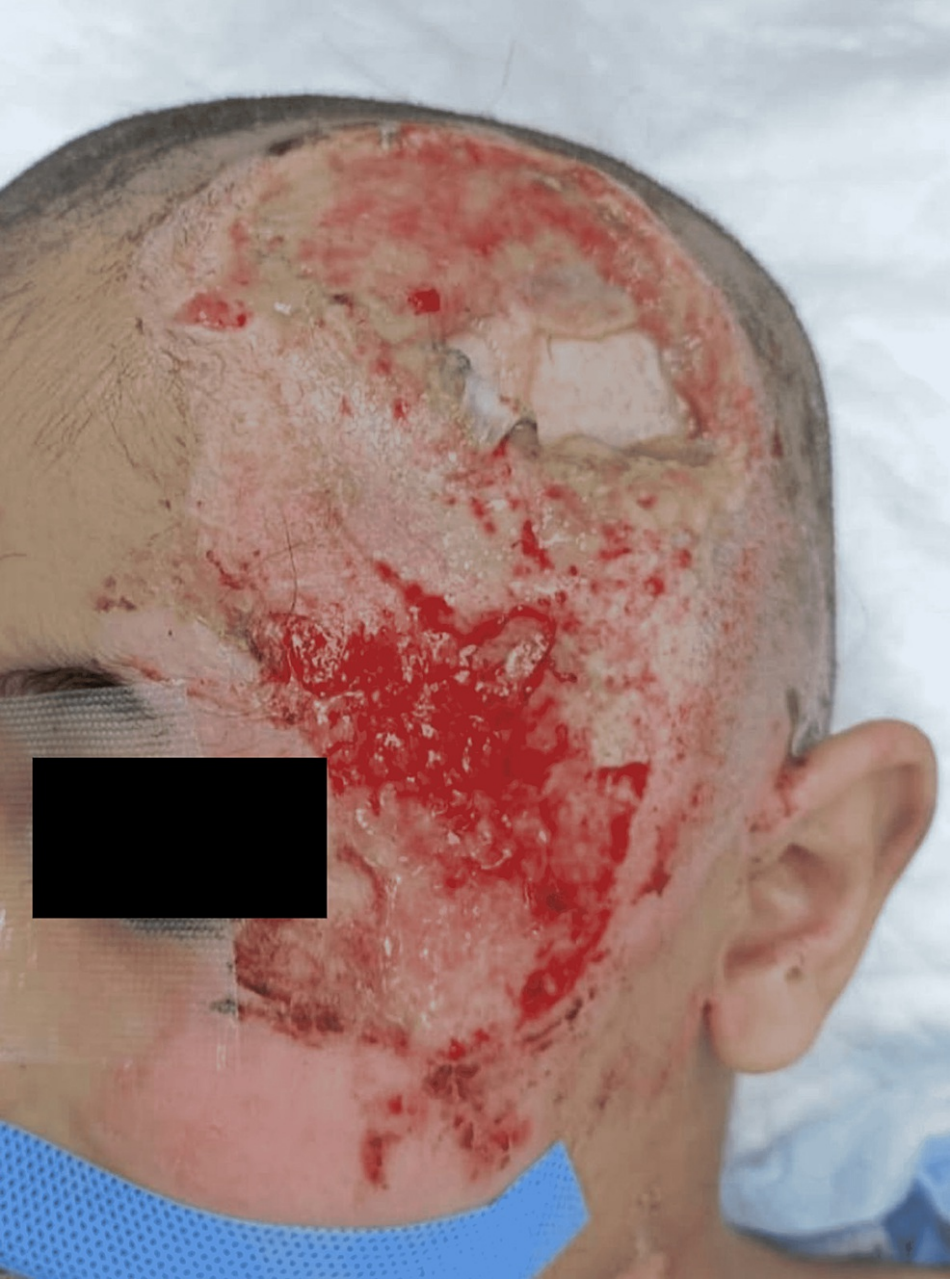
Preoperative photo showing 20 x 10 cm2 scalp defect with exposed cranial bone.

**Figure 2:**
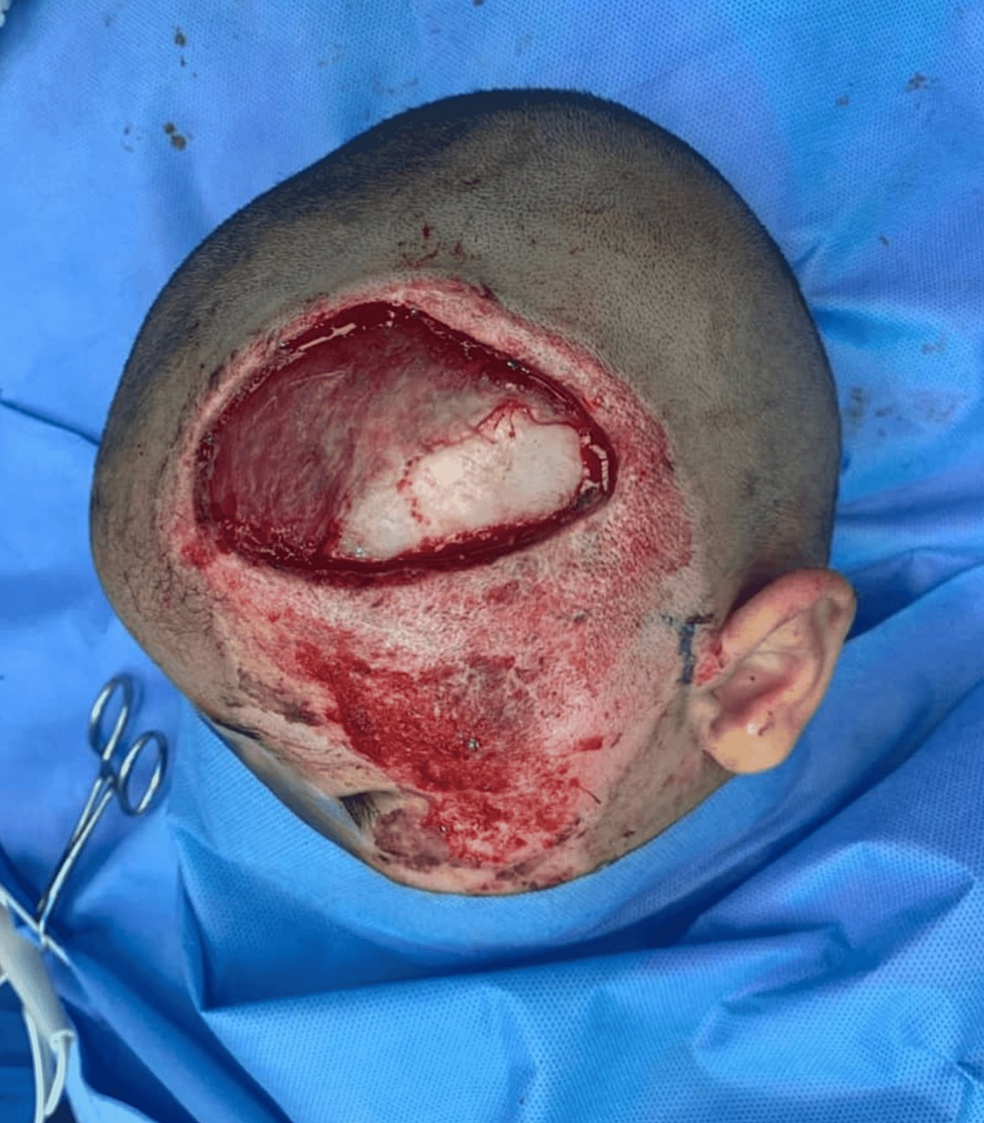
Intraoperative photo showing the defect size after the debridement session.

A 7-year-old boy was referred to our institute five days after a road traffic accident (RTA). The vehicle collided with the pedestrian child. Through friction with the asphalt, he sustained partial to full-thickness defects of the scalp involving the left side of the scalp. He was initially admitted to the pediatric intensive care unit (PICU) in the referring hospital. The preliminary evaluation revealed maintained vital signs, moderate general status, and a preserved Glasgow Coma Scale (GCS) score of 15 with no neurological focal signs or pupillary abnormalities although the computed tomography (CT) scan of the head was showing a small left parietal contusion. The contusion was managed conservatively after a second CT scan demonstrated no progression or new findings. The rest of the history was unremarkable. Upon physical examination, the patient had a large area of full-thickness scalp defect with exposed calvaria surrounded by partial to full-thickness skin loss involving the left temporoparietal region and measuring collectively 20 × 10 cm^2^ in size (Figure 1). In particular, the portion of exposed bone requiring flap coverage was measured after debridement (Figure 2). Inpatient management of the wound consisted of daily moist dressing changes using paraffin gauze containing 0.5% chlorhexidine acetate solution to prevent desiccation of the exposed bone. One day before surgery, the dressing was replaced with a saline-moistened gauze dressing.

**Figure 3:**
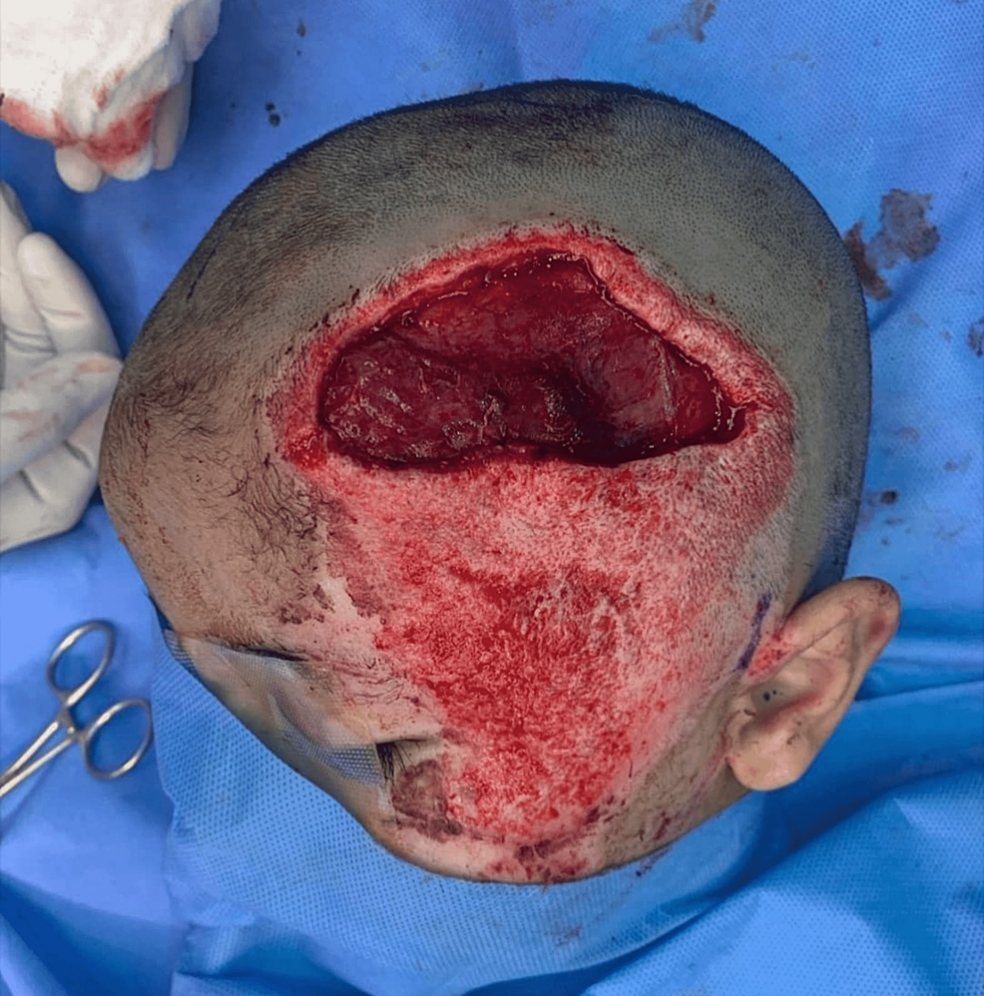
A bipedicled pericranial flap was transposed to cover exposed bone.

**Figure 4:**
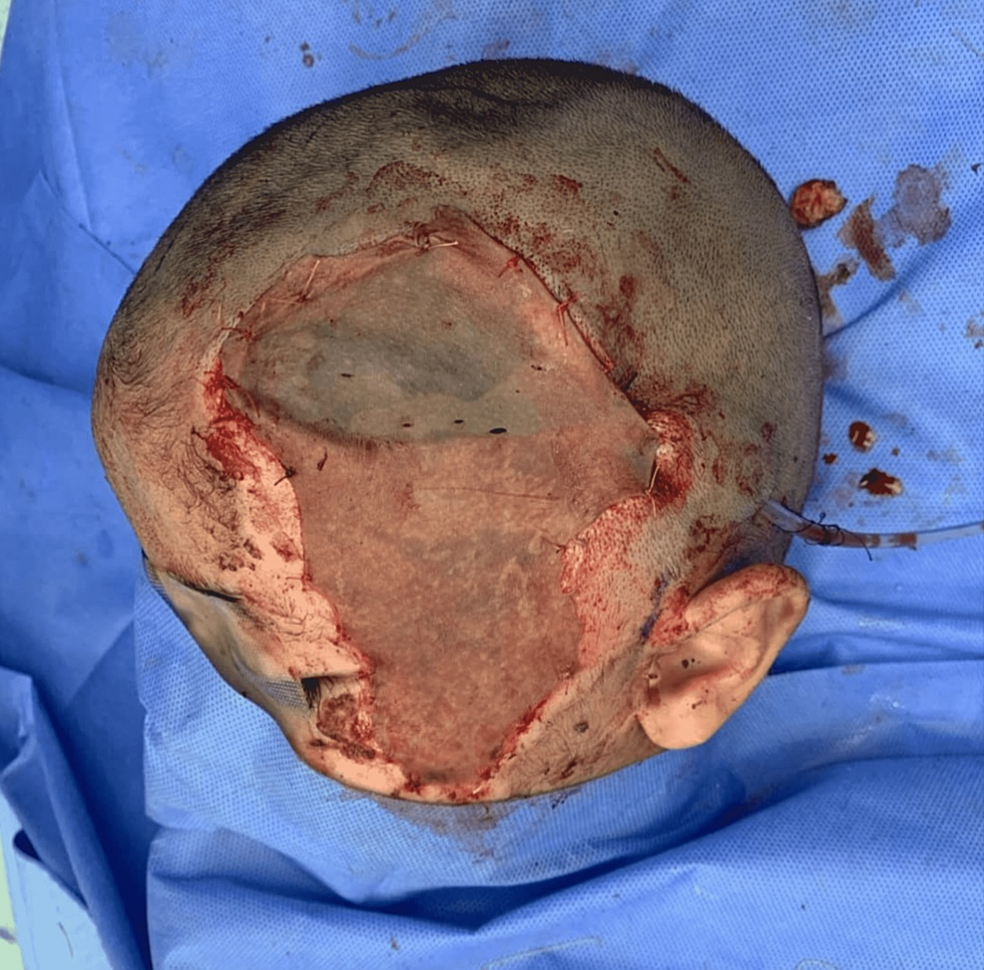
Split-thickness skin graft covering the bipedicled pericranial flap.

**Figure 5:**
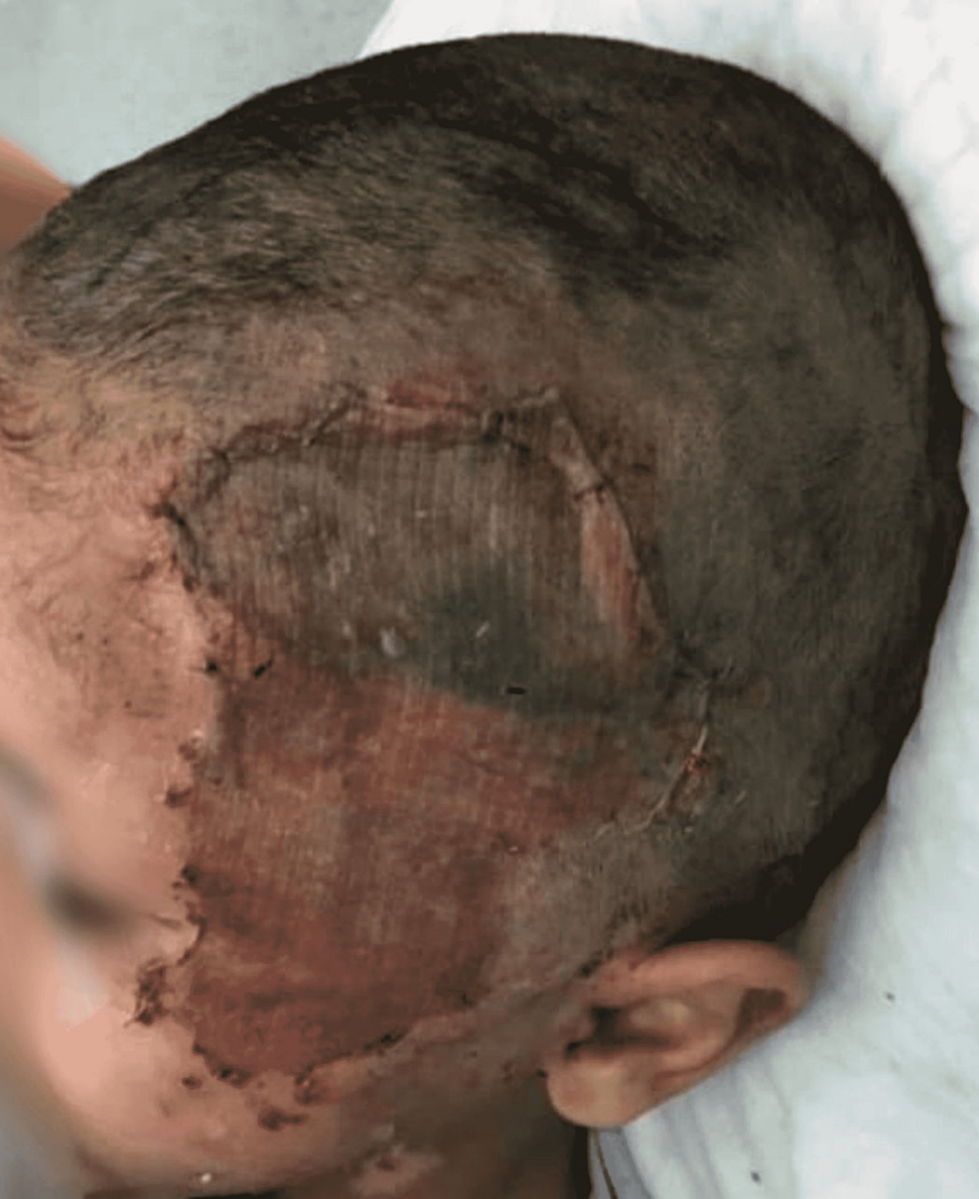
The skin graft taken well on the third postoperative day (POD).

**Figure 6:**
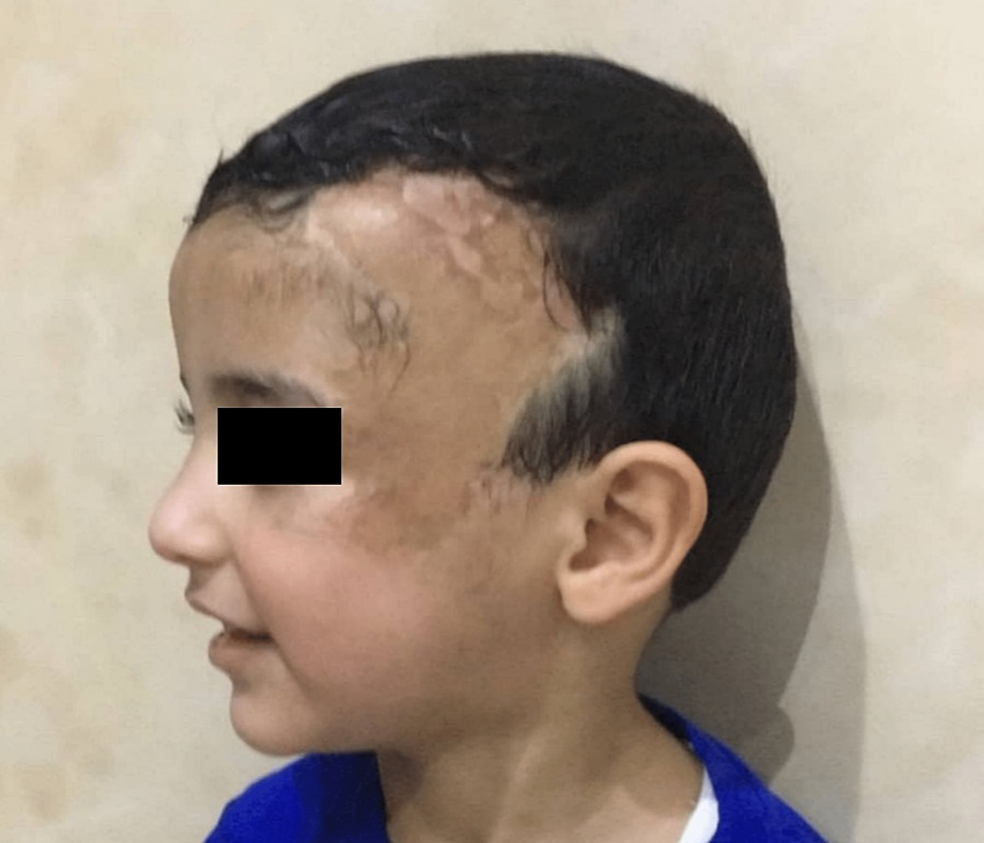
The patient at three months postoperative.

The surgical procedure was performed under general anesthesia with orotracheal intubation. The patient was in a supine position with his head tilted and laying on the right side. The operative site on the left side of the head was facing upwards. The scalp wound and right thigh were disinfected with povidone-iodine 10%. Wound debridement was done, and an 8 × 6 cm soft-tissue defect was created with 5 × 2 cm exposed temporal bone within this defect caudally (Figure 2). The exposed bone was covered by a bipedicled pericranial flap. The pericranial flap was elevated in the subgaleal plane using sharp dissection immediately under the galea aponeurotica. While elevating the flap from the underlying bone, we ensured that adequate subgaleal tissue was attached to the pericranium. A size 15 curved blade was used to release the flap to be transposed to cover the exposed bone. The flap was purely pericranial tissue involving pericranium and subgaleal fascia with no cutaneous component and it was 12 cm long with a pedicle width of 6 cm supplied by supraorbital and supratrochlear vessels anteriorly and post auricular and occipital vessels posteriorly. Bleeding pericranial flap edges were reassuring and demonstrated sufficient vascularity (Figure 3). A size 10 suction drain was placed under the flap to prevent the formation of any underlying hematoma. To ensure the vascularity is not compromised, a tension-free closure was done using absorbable sutures using Vicryl 3-0. Split skin harvested from the right thigh was used to cover the flap and the surrounding raw area (Figure 4). A tie-over bolster dressing was applied. The first dressing was done on the third postoperative day, and the drain was removed. The graft over the flap and surrounding raw area was well taken (Figure 5). The patient was seen at three months postoperatively (Figure 6).

## Discussion

Reconstruction of a scalp defect should ensure the skull’s protection and maintenance of soft-tissue bulk and contour [4]. It should be individualized due to its complexity. When choosing the best option, many factors should be considered. Defect-related factors include size, location and geometry of the defect, quality of the remaining tissue bed and bone, proximity to hardware, and radiation and patient factors such as medical comorbidities and aesthetic outcome [3]. When calvaria is exposed, each option has its own set of advantages and disadvantages. With or without tissue advancement, the primary closure is calvaria burring, followed by grafting, local or regional rotational flap, pedicled flap, and free flap reconstruction [5]. For near-total defects of the scalp, the best reconstructive option is free tissue transfer.

The word pericranium is broadly used for the skull periosteum or the composite of the periosteum and overlying subgaleal fascia [5,6]. We used both the periosteum and subgaleal fascia in our patient making the flap more robust and facilitating easy harvesting than a pure subgaleal fascial flap. Their other advantages include versatility and a lack of functional and morphological complications by avoiding the creation of secondary surrounding skin defects and its associated donor site morbidity preserving this skin to be used for any secondary reconstruction in the future like in resurfacing of the resultant scaring alopecia by skin expansion. Pericranial flaps have been used to support rib grafts in cranial reconstruction by Wolfe [7]. These flaps receive blood supply from major arterial branches of the scalp in an axial pattern from the two pedicles and from the occasional perforators arising from the underlying calvarium ensuring sufficient blood supply to sustain them [8-10]. In contrast, the distal portion of unipedicled pericranial flaps does not have an axial blood supply, and there is no connection with the contralateral pericranial vessels crossing the cranial midline [9]. The vascular supply of the bipedicled pericranial flap depends mainly on the superficial temporal and posterior auricular arteries [7,8]. In our patient, anteriorly the supratrochlear and supraorbital arteries and posteriorly the postauricular and occipital arteries supply the flap. Pericranial flaps have been used for successful reconstruction after trauma or malignancy in dura mater defects, burns, and skin cancer [5-8]. Because these flaps contain both the periosteum and subgaleal fascia, they are usually thick and can find a place in soft-tissue augmentation as a multi-layered flap thereby increasing the bulk and improving the cosmesis in contour deformities [2-3,8].

## Conclusions

This case reports the satisfactory outcomes of using a bipedicled flap in traumatic scalp injuries, specifically in the pediatric age population avoiding the creation of secondary skin defect and its associated donor site morbidity and preserving the skin for any secondary reconstruction in the future. As the flap is bipedicled, it is a morphologically and functionally reliable choice for medium size scalp defects.
